# Differences Between Patients with Probable UIP and Definite UIP on HRCT in Idiopathic Pulmonary Fibrosis: A Real-World Cohort Study

**DOI:** 10.3390/jcm13237170

**Published:** 2024-11-26

**Authors:** Tao Chen, Cheng-Sheng Yin, Ping Wang, Qiu-Hong Li, Chi Shao, Hui Huang, Lan Song, Wei-Hong Zhang, Zuo-Jun Xu

**Affiliations:** 1 Department of Respiratory and Critical Medicine, Peking Union Medical College Hospital, Chinese Academy of Medical Sciences and Peking Union Medical College, No. 1 Shuai Fu Yuan Street, Dong Cheng District, Beijing 100730, China; 2 Department of Respiratory and Critical Medicine, Shanghai Pulmonary Hospital, School of Medicine, Tongji University, Shanghai 200433, China; 3 Department of Radiology, Peking Union Medical College Hospital, Chinese Academy of Medical Sciences and Peking Union Medical College, Beijing 100730, China

**Keywords:** probable usual interstitial pneumonia, idiopathic pulmonary fibrosis, phenotype, prognosis

## Abstract

**Background:** Both a probable usual interstitial pneumonia (UIP) pattern (p-UIP) and a definite UIP pattern (d-UIP) on high-resolution computed tomography (HRCT) are sufficient to establish a diagnosis of idiopathic pulmonary fibrosis (IPF) without the need for a surgical lung biopsy, according to the 2022 IPF guidelines. However, it remains unknown whether patients with p-UIP and d-UIP have similar disease behaviors and clinical courses. **Material and Methods:** We retrospectively collected clinical data of patients with IPF and divided the patients into two groups according to their HRCT patterns: a p-UIP group and a d-UIP group. The baseline characteristics, survival rates, and pulmonary function tests were compared between the two groups. The risk factors for mortality were determined by Cox regression in p-UIP and d-UIP separately. **Results:** There were 304 patients in the p-UIP group and 480 patients in the d-UIP group. Patients in the d-UIP group were more likely to have smoking histories (*p* < 0.001) and had lower baseline FVC% (74% vs. 77%, *p* = 0.021) and DLCO% (50% vs. 58%, *p* < 0.001). Survival rates were higher in p-UIP compared with d-UIP (*p* = 0.004). There were no differences in changes in FVC% or DLCO% between the two groups. Baseline DLCO% was the only independent risk factor for mortality in p-UIP. Baseline FVC% was independently associated with mortality in d-UIP. Symptom of cough was a risk factor for disease progression (OR = 1.2, *p* = 0.002) in p-UIP, while symptom of dyspnea might be associated with disease progression in d-UIP (OR = 2.7, *p* = 0.065). Male patients (OR = 1.88, *p* = 0.002) with a smoking history (OR = 1.16, *p* = 0.002) were at higher risk of developing d-UIP. **Conclusions:** We observed the different disease trajectories between p-UIP and d-UIP. P-UIP on HRCT might identify a subgroup of IPF patients who are in the early stage with a better prognosis.

## 1. Introduction

Idiopathic pulmonary fibrosis (IPF) is characterized by constant progression of fibrosis and persistent decline of lung function [1]. It is extensively studied due to its poor prognosis with a median survival time of 2–4 years [2]. According to the 2022 IPF guidelines [2], IPF patients are divided into four groups by their high-resolution chest computed tomography (HRCT) patterns: definite usual interstitial pneumonia (d-UIP), probable UIP pattern (p-UIP), indeterminate for UIP, and alternative diagnosis. A d-UIP pattern on HRCT is sufficient to establish a diagnosis of IPF without the need for a surgical lung biopsy. The evidence has indicated that patients with indeterminate UIP have higher lung function and a better prognosis than those with p-UIP and d-UIP [3].

According to the latest guidelines, most patients with p-UIP on HRCT can be diagnosed with IPF, unless a lung biopsy suggests an alternative diagnosis [2]. Studies suggested that 80–85% of patients with probable UIP on HRCT were confirmed with UIP in histological findings [4,5]. And the coexistence of reticular and traction bronchiectasis on HRCT increased the positive predictive value of histopathologically proven UIP [6]. Therefore, in multicenter clinical trials of IPF, p-UIP on HRCT was practically diagnosed with IPF without a surgical lung biopsy [7].

Some researchers were considering merging d-UIP and p-UIP patterns in one group. There were different opinions about whether patients with p-UIP and d-UIP have similar disease behaviors and clinical courses. Some studies found that patients with p-UIP had similar lung function changes with a d-UIP pattern [3,8]. But others found that p-UIP had longer Kaplan–Meier event-free survival and lower rates of acute exacerbations of IPF than d-UIP [9,10,11]. Therefore, a consensus on this issue has not been reached.

In this study, we divided patients with IPF into two groups according to their HRCT patterns—a p-UIP group and a d-UIP group—and compared the survival rates and trajectories of lung function changes and explored risk factors for mortality and disease progression.

## 2. Methods

**Figure 1:**
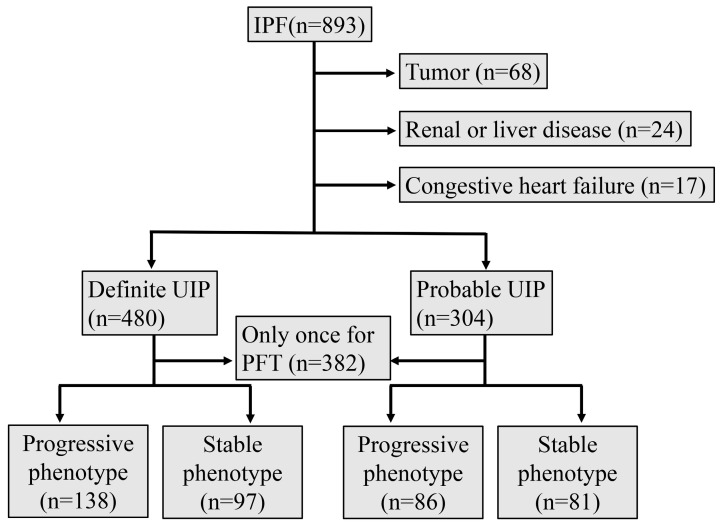
The flowchart of the study. IPF, idiopathic pulmonary fibrosis; PFT, pulmonary function test; UIP, usual interstitial pneumonia.

We retrospectively collected clinical data of IPF patients with at least one pulmonary function test and HRCT who were diagnosed in Peking Union Hospital from 1 January 2013 to 1 January 2024. Patients with tumors, severe renal or liver diseases, and congestive heart failure were excluded. Finally, there were 784 patients included in this study (Figure 1). The study was approved by the Ethics Committee of the Chinese Academy of Medical Sciences and Peking Union Medical College Hospital (JS-1127). Informed consent to participate in the study was obtained from all participants.

### 2.1. Diagnostic Criteria

IPF was diagnosed according to the latest version of the guidelines [2]. Given the retrospective nature of the study, the multidisciplinary board in our center reviewed follow-up data and performed the final evaluation to determine the definitive clinical diagnosis. Once the patients were diagnosed with IPF, they were treated with antifibrotic drugs on the basis of their agreement. Two experienced radiologists (SL and ZWH) reviewed the HRCT at the time of diagnosis and classified patients into definite UIP and probable UIP according to the characteristics of the imaging. p-UIP was defined as a subpleural and basal predominant reticular pattern with traction bronchiectasis or bronchiolectasis. d-UIP was defined as subpleural and basal predominant honeycombing with or without traction bronchiectasis. The classical HRCT images are shown in Figure S1. The controversial cases were discussed until a consensus was reached. For further analysis, patients in the probable UIP group were divided into two subgroups: the progressive phenotype and the stable phenotype. Disease progression was defined by the diagnostic criteria of progressive pulmonary fibrosis from the 2022 IPF guidelines [12]. Specifically, the progressive phenotype was defined as at least two of the following three criteria occurring within one year with no alternative explanation: (1) worsening respiratory symptoms; (2) physiological evidence of disease progression; and (3) radiological evidence of disease progression.

### 2.2. Data Collection

The survival status, lung transplantation events, and causes of death from diagnosis to the end of follow-up were obtained from electronic medical records and/or telephone interviews. The follow-up duration was determined as the time from the initial clinical visit in Peking Union Hospital to death or the censoring date of 1 January 2023 for survivors. Demographic characteristics, smoking history, symptoms (including cough and apnea), pulmonary function tests (PFTs), resting oxygen saturation, serum autoantibodies, and oxygen therapy status were collected from the electronic medical record system. Some patients had records of cough VAS, six-minute-walking-distance (6MWD), and exertional oxygen saturation. Spirometry was conducted according to the recommendations of the Chinese Thoracic Society (CTS). Only maneuvers of CTS quality grade C or above were used. Raw values of FEV1 and FVC were converted to percent predicted based on a previously published lung function prediction equation [13].

### 2.3. Statistical Analysis

The enumeration data were presented as numbers (percents). Quantitative data were presented as means ± standard deviations or medians (interquartile ranges) if they did not conform to a normal distribution. For normally distributed data, an independent-sample *t*-test was used for two-group comparisons. A chi-square test was performed to compare the rates. *p* < 0.05 was used to indicate statistical significance.

Kaplan–Meier and log-rank tests were used to compare the survival times between the two groups. The survival time was defined from the date of diagnosis to the date of death or lung transplantation. Patients were censored at the time of the last clinic visit. Cox regression was performed to determine risk factors for mortality. First, univariate Cox analyses were performed, followed by multivariate Cox analysis using the significant risk factors (*p* < 0.05) in the univariate analysis.

All the PFT analyses were carried out on patients with at least two PFTs. The changes in FVC% and DL_CO_% were estimated by a linear mixed-effects model, in which the fixed effects included the time interval for the PFT measurement, the HRCT pattern, age, sex, and baseline FVC% and DL_CO_%. Then, the predicted values of FVC% change and DL_CO_% change were fitted by linear regression. Given the retrospective nature of this study, the PFT results were collected from the electronic medical record system, and the time intervals of the PFTs were different among patients, which limited us to comparing the PFT parameters at several fixed time points, such as 3, 6, and 12 months. The linear mixed-effects model enabled us to include all the PFT results at different time points and fit a line for the change in FVC and DLCO.

Multivariate logistic regression was used to determine the risk factors for disease progression in patients with probable UIP and to determine the risk factors for developing d-UIP. Patients with a 12-month PFT re-examination were included in this analysis. To remove potential bias, patients with and without a 12-month PFT were compared (Table S1). All analyses were performed using Stata version 16 (StataCorp LLC, College Station, TX, USA).

## 3. Results

### 3.1. Baseline Data

There were 304 patients in the p-UIP group and 480 patients in the d-UIP group (Table 1). No differences were observed in age, sex, body mass index (BMI), or rates of oxygen therapy between the two groups. Patients in the d-UIP group were more likely to have a smoking history (66.4% vs. 79.2%, *p* < 0.001) with a higher number of smoking pack-years (26 vs. 20, *p* = 0.090). Patients in the d-UIP group had lower baseline FVC% (74% vs. 77%, *p* = 0.021) and DLCO% (50% vs. 58%, *p* < 0.001) and lower resting oxygen saturation (96.4% vs. 97.2%, *p* = 0.006). Patients with p-UIP were different from those with d-UIP in terms of smoking history and PFT.

There were 224 patients in the progressive group and 178 patients in the stable group. No differences were observed in age, sex, or pulmonary function tests between the two groups. Patients in the progressive group had severe symptoms of cough (*p* < 0.001) and dyspnea (*p* = 0.003), and patients in the progressive group had higher rates of oxygen therapy (*p* = 0.001) but lower rates of treatment with antifibrotic drugs (*p* < 0.001). When we compared d-UIP with progressive p-UIP, we observed similar results (Table S2). Patients with progressive phenotypes had similar PFT results but severe symptoms when compared with patients in the stable group.

### 3.2. Comparison of Progressive Subgroup with Stable Subgroup in Patients with Probable UIP and Definite UIP

Next, we explored the differences between the progressive and stable groups with d-UIP and p-UIP, respectively. There were 167 patients and 235 patients with follow-up data in the p-UIP and d-UIP groups, respectively. A total of 86 patients were identified as progressive p-UIP and 81 patients were identified as stable p-UIP, while 138 patients were identified as progressive d-UIP and 97 patients were identified as stable d-UIP (Table 2). There was a numerical but not statistically significant decrease in 6MWD (d-UIP: 400 vs. 449, *p* = 0.089; p-UIP: 402 vs. 457, *p* = 0.079) in both progressive groups. Interestingly, both progressive phenotypes had severe cough symptoms (d-UIP: 35.7 vs. 26.9, *p* = 0.024; p-UIP: 35.8 vs. 23.4, *p* = 0.001). Additionally, the progressive p-UIP subgroup had higher rates of dyspnea (55.8% vs. 34.6%, *p* = 0.003) and higher rates of oxygen therapy (38.4% vs. 18.5%, *p* = 0.003) compared with the stable p-UIP subgroup (Table S3). Conclusively, symptoms seem to be more relevant to disease progression than to PFT.

### 3.3. Survival Rates

**Figure 2:**
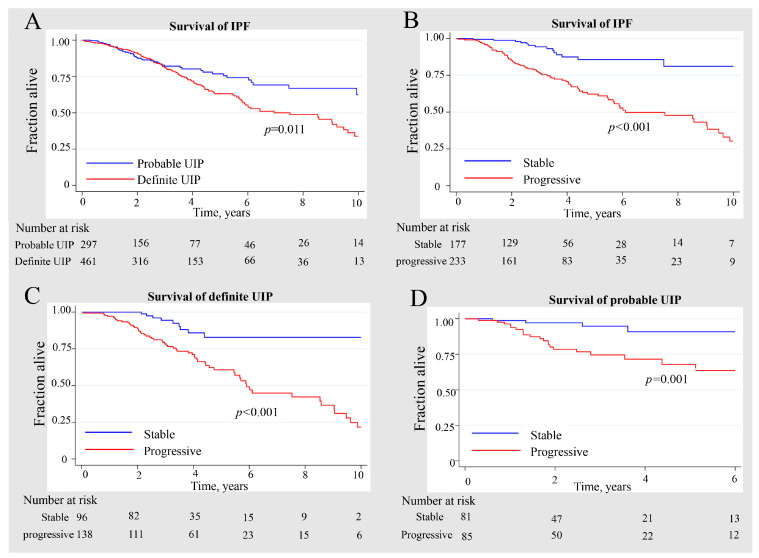
The survival of the different IPF groups. (**A**) Survival curves of patients with p-UIP and d-UIP. (**B**) Survival curves of IPF patients with progressive and stable phenotypes. (**C**) Survival curves of d-UIP patients with progressive and stable phenotypes. (**D**) Survival curves of p-UIP patients with progressive and stable phenotypes. d-UIP, definite usual interstitial pneumonia; p-UIP, probable usual interstitial pneumonia.

The Kaplan–Meier method was used to estimate survival times (Figure 2). Within 10 years of follow-up, patients with p-UIP had a significantly higher survival rate than those with d-UIP (*p* = 0.011). The 5-year survival rates were 76.8% (95% CI: 69.1%-82.9%) for p-UIP and 63.1% (95% CI: 56.7%-68.8%) for d-UIP (Figure 2A). For further analysis, in male and never-smoker subgroups, p-UIP had a higher survival rate than d-UIP (Figures S2 and S3). Next, we compared survival rates between the progressive phenotype and the stable phenotype in all IPF patients, p-UIP and d-UIP. The progressive group had lower survival rates than that of the stable group (Figure 2B–D; IPF: *p* < 0.001; d-UIP: *p* < 0.001; p-UIP: *p* = 0.001). Overall, patients with p-UIP had higher survival rates than those with d-UIP, and both p-UIP and d-UIP had stable phenotypes, which had significantly better survival than the progressive phenotype.

### 3.4. Risk Factors for Mortality

Next, we used the Cox regression model to identify the risk factors for mortality (Table 3). In univariate analysis, lower baseline FVC% and DL_CO_%, higher cough VAS, having symptoms of dyspnea, and needing oxygen therapy were identified as the risk factors for mortality in both p-UIP and d-UIP. The above factors and age were taken into the multivariable model; only higher baseline DL_CO_% was independently associated with a reduced hazard of death or lung transplantation for p-UIP (HR = 0.60, 95% CI: 0.40–0.89; *p* = 0.011). In patients with d-UIP, only baseline FVC% (HR = 0.85, 95% CI: 0.77–0.95; *p* = 0.003) was identified as an independent risk factor for mortality. When we merged patients with d-UIP and progressive p-UIP, both baseline DLCO% and FVC% were associated with mortality (Table S4).

### 3.5. Pulmonary Function Test

**Figure 3:**
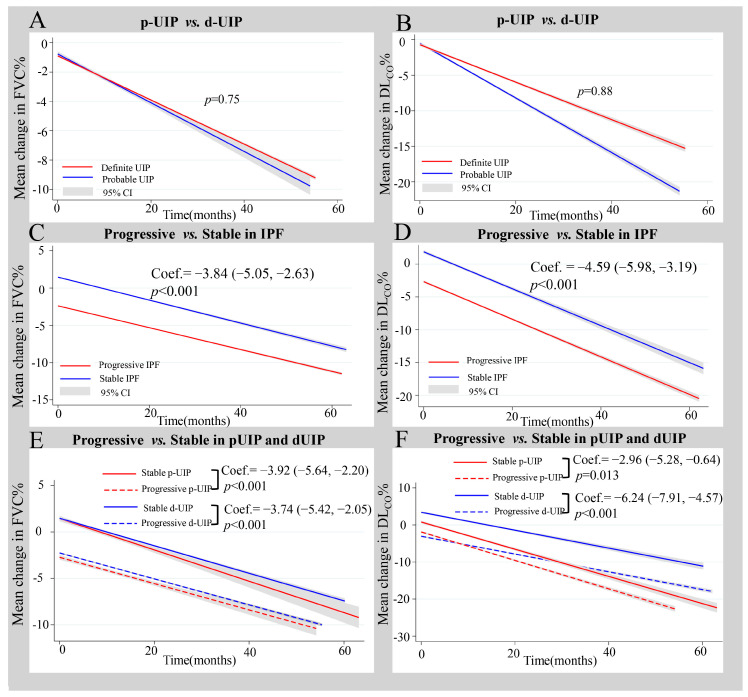
The changes in pulmonary function parameters in the different groups of IPF (**A**) The changes in FVC% between d-UIP and p-UIP. (**B**) The changes in DL_CO_% between d-UIP and p-UIP. (**C**) The changes in FVC% between progressive IPF and stable IPF. (**D**) The changes in DL_CO_% between progressive IPF and stable IPF. (**E**) The changes in FVC% between progressive and stable phenotypes of d-UIP and p-UIP. (**F**) The changes in DL_CO_% between progressive and stable phenotypes of d-UIP and p-UIP. DL_CO_, diffusing capacity of the lungs for carbon monoxide; d-UIP, definite UIP; FVC, forced vital capacity; p-UIP, probable UIP.

In the mixed-effects model (Figure 3), after adjusting for age, gender, baseline FVC and DL_CO_, and interval of PFT time, the HRCT pattern (p-UIP vs. d-UIP) was not associated with the changes in FVC% and DL_CO_% (Figure 3A,B; FVC: *p* = 0.75; DL_CO_: *p* = 0.88). For patients with definite UIP, the average annual changes in FVC% and DL_CO_% were −1.82 (95% CI: −2.27, −1.38) and −3.05 (95% CI: −3.57, −2.52). For patients with p-UIP, the annual changes in FVC% and DL_CO_% were −2.18 (95% CI: −2.81, −1.55) and −4.45 (95% CI: −5.31, −3.60). In the subgroup analysis, female p-UIP patients exhibited a greater decline in FVC% compared with female d-UIP patients (Table S5; *p* = 0.041).

Compared with stable IPF, progressive IPF exhibited a greater decrease in FVC% [Figure 3C; −3.84, 95% CI: (−5.05, −2.63), *p* < 0.001] and DL_CO_% [Figure 3D; −4.59, 95% CI: (−5.98, −3.19), *p* < 0.001]. Moreover, in the p-UIP and d-UIP subgroups, the progressive phenotype also exhibited a greater decrease in FVC% (Figure 3E; p-UIP, *p* < 0.001; d-UIP, *p* < 0.001) and DL_CO_% (Figure 3F; p-UIP, *p* = 0.013; d-UIP, *p* < 0.001) than the stable phenotype. Above all, changes in FVC% and DL_CO_% were similar in p-UIP and d-UIP, but the stable phenotype exhibited a milder decrease in pulmonary function than the progressive phenotype.

### 3.6. Risk Factors for Disease Progression in p-UIP

**Figure 4:**
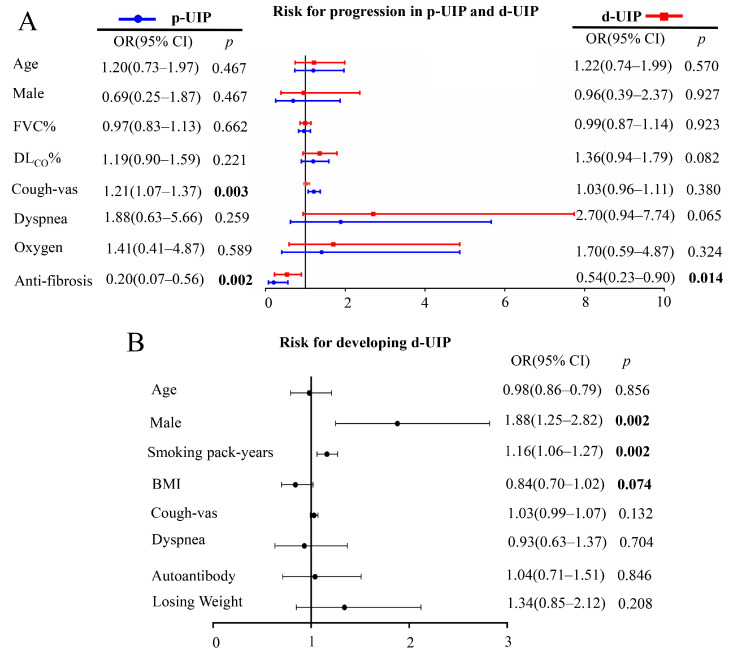
Risk factors for progression in p-UIP and d-UIP (**A**) and developing d-UIP (**B**). We evaluated the impact of increasing age by 10 years, smoking pack-years by 10, BMI by 5, and cough VAS by 5 units. The bold indicates a statistical significance. BMI, body mass index; DL_CO_, diffusing capacity of the lungs for carbon monoxide; d-UIP, definite usual interstitial pneumonia; FVC, forced vital capacity; p-UIP, probable usual interstitial pneumonia.

Multivariate logistic regressions were performed to identify factors associated with disease progression in p-UIP and d-UIP (Figure 4A). Anti-fibrosis treatments were associated with decreased risks of disease progression in both p-UIP [OR: 0.20, 95% CI: (0.07–0.56), *p* = 0.002] and d-UIP [OR: 0.54, 95% CI: (0.23–0.90), *p* = 0.014]. For patients with probable UIP, cough VAS was a risk factor for disease progression (OR: 1.2, 95% CI: (1.07–1.35), *p* = 0.002). For patients with definite UIP, symptoms of dyspnea might be a risk factor for disease progression (OR: 2.70, 95% CI: (0.94–7.74), *p* = 0.065).

### 3.7. Risk Factors for Developing d-UIP

Multivariate logistic regression was performed to determine the risk factors for developing d-UIP. After adjusting for age, auto-antibodies, and symptoms, male gender (OR: 1.88, 95% CI: (1.25–2.82), *p* = 0.002) and more smoking pack-years (OR: 1.16, 95% CI: (1.06–1.27), *p* = 0.002) were risk factors for developing d-UIP (Figure 4B). Interestingly, a higher BMI seemed to be a protective factor for developing d-UIP (OR: 0.84, 95% CI: (0.70–1.02), *p* = 0.074). In conclusion, male patients with a smoking history were at high risk of developing d-UIP.

## 4. Discussion

From our clinical observations, the p-UIP pattern has a poorer prognosis compared with other patterns on HRCT, like cryptogenic organizing pneumonia (COP) [14]. But in patients with IPF, the p-UIP pattern implies an early stage, which means that some patients with p-UIP will experience a stable period of disease course [15]. Therefore, it is vital to delineate the disease trajectory and explore the risk factors for progression in p-UIP. In this study, we found no differences in the decline in FVC% and DL_CO_% between p-UIP and d-UIP. However, p-UIP had lower mortality compared with d-UIP. The independent risk factors for mortality for p-UIP and d-UIP were lower baseline DL_CO_% and FVC%, respectively. For p-UIP, the predictor of disease progression was more severe symptoms of cough (Figure S5). Higher numbers of smoking pack-years and male gender were risk factors for developing d-UIP in IPF. Our results first described the survival rates and changes in pulmonary function for p-UIP and d-UIP in a real-world cohort treated with anti-fibrosis drugs, and we comprehensively evaluated the risk factors for developing d-UIP, disease progression, and mortality in the two groups.

### 4.1. Differences Between p-UIP and d-UIP

From our results, we considered p-UIP as a separate phenotype of IPF, different from d-UIP. First, patients with p-UIP had lower mortality rates than those with d-UIP. Similar results were also found in other studies. Gaya and colleagues found an increased survival rate of 30% in p-UIP compared with d-UIP [16]. Other studies also demonstrated a longer median survival time in p-UIP (62.5–72.1 months) compared with d-UIP (42.8–43.5 months) [9,10]. The higher survival rates mean a better prognosis in p-UIP.

Secondly, patients with d-UIP had lower baseline pulmonary functions and more severe symptoms of dyspnea and cough. These results further confirmed our clinical observation that d-UIP always represents a late phase of the disease. The possible explanation is that d-UIP lesions are histologically stiffer and therefore have a greater influence on lung function compared with p-UIP. The supporting data were also reported in other studies. Researchers observed worse pulmonary function in d-UIP [3,9]. Additionally, male gender and smoking history might be risk factors for developing d-UIP. Similar results were reported in another study, in which male gender increased the probability of histological confirmation of UIP in patients with p-UIP [6].

Thirdly, d-UIP and p-UIP had different risk factors for mortality. When we put FVC% and DLCO% into the multivariable model, higher baseline FVC% was associated with decreased mortality in d-UIP, while higher baseline DLCO% was associated with decreased mortality in p-UIP. Our results were consistent with the GAP staging system, which included both FVC% and DLCO% to evaluate the prognosis [17,18]. We postulated that the reason might be that the decrease in FVC% was irreversible and progressive for d-UIP [19]. However, our further analysis illustrated that a subgroup of p-UIP patients had a significantly slower decline in FVC% compared with d-UIP, after dividing patients with p-UIP into progressive and stable subgroups. Additionally, a proportion of patients with p-UIP progressed, and some p-UIP patients transformed into d-UIP during the disease progression. A previous study included 68 patients with p-UIP and 32 (47%) transformed into d-UIP with disease progression [20]. In our cohort, we divided patients with progressive p-UIP into three groups (Figure S4): (a) p-UIP progressed into d-UIP (*n* = 6, 7.0%); (b) p-UIP progressed with honeycomb formations in some areas (*n* = 17, 19.8%); (c) p-UIP progressed with no honeycomb formations (*n* = 63, 73.3%). About 26.7% of progressive p-UIP patients transformed into d-UIP in our cohort.

Lastly, only symptom of cough was a predictor of disease progression in p-UIP, which indicated that symptoms preceded the decline in pulmonary function in p-UIP. Unlike the patients with d-UIP, who developed mature pathological lesions with honeycombing containing more collagen, patients with p-UIP might have had more inflammatory contextures in the pulmonary fibrotic areas [21]. These differences could have led to a synchronous change between FVC% and symptoms in d-UIP. On the contrary, p-UIP lesions caused severe symptoms of cough, as they contained more inflammatory infiltrates and cytokines, which stimulated the cough reflex [22]. As a result, more severe symptoms indicated more severe inflammatory reactions, thereby predicting the decline in pulmonary function. That explained why we observed more severe symptoms of cough and dyspnea in progressive p-UIP compared with stable p-UIP, which was also demonstrated in another study [23]. Previous studies also illustrated that cough was a predictor of IPF progression independently of disease severity [24], which might have been due to a profibrotic feedback loop. Mechanistically, cough in IPF might be produced by chemical stimulation of peripheral cough receptors. These chemical signals might be stimulated by inflammatory cytokines, which proved to be increased in BALF from patients with IPF [25,26]. In turn, cough, as an additional source of mechanical stretching, might activate the pro-fibrotic pathway in lung tissues in IPF [27]. The above results remind clinicians to pay attention to symptom evaluation and management, especially for patients with p-UIP. Additionally, it is unknown whether neurotransmitter factors account for the disease progression in these patients.

Conclusively, IPF, recognized as a progressive disease, was stratified into different phenotypes. And it might be a promising research field to explore the different therapeutic strategies in different phenotypes. For example, although corticosteroids and immunosuppressants have been demonstrated to be ineffective in IPF [28], it is unclear whether these drugs or targeted anti-inflammatory agents are beneficial for patients with p-UIP.

### 4.2. The Similarity Between p-UIP and d-UIP

Our results and former studies together demonstrated that rates of decline in FVC% and DL_CO_% were similar between p-UIP and d-UIP [3]. Raghu and colleagues found that IPF with d-UIP and p-UIP had a similar annual decline in FVC, which amounted to 220 mL in the antifibrotic group and 110 mL in the placebo group [8]; they also found that patients with p-UIP responded similarly to patients with d-UIP to Nintedanib. Another study found that lung functions were correlated with the extent of radiological disease but not specific morphologic patterns of UIP [29]. These results demonstrated the nature of the progressive decline in pulmonary function in IPF and confirmed the accuracy of the diagnosis [15]. Nevertheless, we demonstrated the heterogeneity in p-UIP, having obtained clues from survival curves. The first two years’ survival curve trends were quite different from those of the following years in p-UIP. In the beginning, we postulated that patients with lower baseline pulmonary function had a worse prognosis, as previous studies had suggested [30]. Surprisingly, those with the progressive phenotype had similar baseline pulmonary function to those with the stable phenotype. Our results provided the cues to discriminate progressive and stable p-UIP by symptom severity. But further research is needed to explore sera or BALF signatures between the two groups.

In this research, the baseline pulmonary functions were the risk factors for mortality in both p-UIP and d-UIP. In previous research, DLCO% and FVC% were broadly recognized as risk factors for mortality, acute exacerbations, and disease progression in fibrotic interstitial lung diseases and progressive pulmonary fibrosis [31,32,33]. These results indicate the importance of regular monitoring of lung function for IPF patients.

### 4.3. Limitations

Our research was based on a large real-world cohort with a sample size of about 900 IPF patients. Additionally, there was no loss of data in the survival analysis. These factors guaranteed the objectivity of the results. However, there were also some limitations. First, we did not perform a sensitivity analysis. Fortunately, the subgroup analysis was conducted throughout our study, which might have compensated for the lack of a sensitivity analysis. Secondly, a relatively short median follow-up time prevented us from obtaining a reliable survival time, but it did not influence the comparison of survival rates between the two groups. Thirdly, this was a retrospective study, and the median follow-up time of our cohort was 3.6 years, which might have influenced the comparison of our results with those of other IPF cohorts. For example, the median survival time of our cohort was over six years; however, previous studies showed a median survival time of less than 5 years [9,32]. In addition, we were also limited to capturing long-term outcomes of the two groups of patients (p-UIP and d-UIP), especially in the evaluation of estimated pulmonary function. Therefore, further prospective research with a longer follow-up time is needed to verify the results. Lastly, in our cohort, the differences in baseline FVC% and DL_CO_% between p-UIP and d-UIP might have influenced the survival rates. Fortunately, d-UIP only exhibited a decrease of 3% in FVC% compared with p-UIP. A prospective clinical study is needed to further validate our results.

## 5. Conclusions

We observed the different disease trajectories of p-UIP and d-UIP. P-UIP on HRCT might identify a subgroup of IPF patients in the early stage with a better prognosis. Further research is needed to explore the different mechanisms between d-UIP and p-UIP.

## Figures and Tables

**Table 1:** The baseline characteristics of patients with probable UIP compared with those of patients with definite UIP.

Variables	Progressive	Stable	*p*	Probable UIP	Definite UIP	*p*
Sample size	224	178		304	480	
Age, years	69 ± 8	67 ± 9	0.109	68 ± 9	69 ± 8	0.547
Male sex	166 (74.1%)	127 (71.3%)	0.537	215 (70.7%)	363 (75.6%)	0.129
BMI	28.7 ± 4.9	28.3 ± 4.6	0.383	28.4 ± 5.0	28.4 ± 5.0	0.850
Ever-smoker	158 (71.5%)	130 (73.9%)	0.599	202 (66.4%)	380 (79.2%)	<0.001
Smoking pack-years	25 [12–40]	26 [11–36]	0.961	20 [9–36]	26 [13–41]	0.090
FVC, L	2.8 ± 0.8	2.8 ± 0.8	0.887	2.8 ± 0.9	2.8 ± 0.8	0.238
FVC, % predicted	75.4 ± 19.1	75.8 ± 18.1	0.841	77 ± 19	74 ± 19	0.021
DLCO, % predicted	53.8 ± 17.0	55.8 ± 18.3	0.339	58 ± 18	50 ± 16	<0.001
6MWD	401 ± 142	456 ± 102	0.013	419 ± 131	410 ± 131	0.618
Resting oxygen saturation	97.3 ± 2.1	96.9 ± 1.9	0.222	97.2 ± 2.0	96.4 ± 2.8	0.006
Exertional oxygen saturation	87.4 ± 6.3	88.1 ± 5.6	0.530	88.4 ± 6.0	88.3 ± 5.8	0.857
Oxygen therapy	69 (35.6%)	33 (20.1%)	0.001	73 (24.0%)	102 (21.3%)	0.820
Cough	158 (84.0%)	115 (77.7%)	0.139	197 (64.8%)	328 (68.3%)	0.306
Cough VAS	32 [13–55]	17 [7–35]	<0.001	21 [9–46]	26 [10–52]	0.097
Dyspnea	119 (53.1%)	68 (38.2%)	0.003	149 (49.0%)	262 (54.6%)	0.074
Weight loss	40 (19.4%)	38 (23.9%)	0.300	46 (15.1%)	109 (22.7%)	0.009
Auto-antibodies	84 (37.5%)	59 (33.1%)	0.365	96 (31.6%)	173 (36.0%)	0.114
GERD	62 (30.0%)	40 (25.3%)	0.328	74 (24.3%)	108 (22.5%)	0.305
Anti-fibrotic drugs	106 (43.7%)	124 (72.7%)	<0.001	165 (59.6%)	262 (69.1%)	0.011

Abbreviations: 6MWD, 6 min walking distance; BMI, body mass index; DLCO, diffusing capacity of the lungs for carbon monoxide; FVC, forced vital capacity; GERD, gastroesophageal reflux disease; UIP, usual interstitial pneumonia.

**Table 2:** The baseline characteristics of patients with progressive and stable phenotypes in p-UIP and d-UIP.

Variables	Definite UIP			Probable UIP		
	Progressive	Stable	*p*	Progressive	Stable	*p*
Sample size	138	97		86	81	
Age, years	69.4 ± 7.1	67.6 ± 8.7	0.085	67.8 ± 9.4	67.2 ± 8.6	0.692
Male sex	108 (78.3%)	70 (72.2)	0.283	58(67.4%)	57 (70.4%)	0.683
BMI	28.7 ± 4.6	28.7 ± 4.8	0.962	28.7 ± 5.5	27.8 ± 4.2	0.244
Ever-smoker	103 (74.6%)	79 (81.4%)	0.219	55 (66.3%)	51 (64.6%)	0.819
Smoking pack-years	25 [14–41]	26 [8–38]	0.850	20 [7–37]	21 [13–36]	0.926
FVC, L	2.8 ± 0.8	2.7 ± 0.8	0.243	2.7 ± 0.9	2.9 ± 0.9	0.161
FVC, % predicted	75 ± 18	74 ± 18	0.779	76 ± 20	78 ± 18	0.659
DLCO, % predicted	51 ± 16	50 ± 16	0.552	58 ± 18	63 ± 19	0.125
6MWD	400 ± 137	449 ± 105	0.089	402 ± 154	457 ± 104	0.079
Resting oxygen saturation	97.3 ± 2.2	96.2 ± 1.7	0.013	97.3 ± 2.0	97.7 ± 1.8	0.306
Exertional oxygen saturation	87.2 ± 6.2	87.8 ± 5.1	0.653	87.7 ± 6.5	88.3 ± 6.0	0.696
Cough VAS	35.7 ± 29.1	26.9 ± 24.6	0.024	35.8 ± 15.2	23.4 ± 12.1	0.001
Dyspnea	71 (51.4%)	41 (42.3%)	0.165	48 (55.8%)	28 (34.6%)	0.003
Oxygen therapy	36 (32.1%)	17 (20.2%)	0.063	33 (38.4%)	15 (18.5%)	0.003

Abbreviations: 6MWD, 6 min walking distance; BMI, body mass index; DL_CO_, diffusing capacity of the lungs for carbon monoxide; FVC, forced vital capacity; UIP, usual interstitial pneumonia.

**Table 3:** Risk factors associated with survival in patients with probable and definite UIP.

**Probable UIP**				
**Variables**	Univariate *		Multivariate *	
	**HR (95% CI)**	***p*-Value**	**HR (95% CI)**	***p*-Value**
Age	0.86 (0.56–1.32)	0.484	0.55 (0.26–1.14)	0.106
Male	0.95 (0.43–2.12)	0.899		
BMI	1.00 (0.93–1.08)	1.000		
Smoked	1.20 (0.52–2.74)	0.673		
FVC%	0.77 (0.69–0.86)	<0.001	1.00 (0.84–1.17)	0.953
DLCO%	0.50 (0.36–0.69)	<0.001	0.60 (0.40–0.89)	0.011
Cough VAS	1.15 (1.08–1.23)	<0.001	1.05 (0.94–1.16)	0.323
Dyspnea	3.57 (1.56–8.17)	0.003	0.90 (0.18–4.50)	0.900
Oxygen therapy	7.53 (3.14–18.1)	<0.001	3.02 (0.61–15.01)	0.176
**Definite UIP**				
**Variables**	**Univariate**		**Multivariate**	
	**HR (95% CI)**	***p*-Value**	**HR (95% CI)**	***p*-Value**
Age	1.05 (0.85–1.29)	0.674	1.08 (0.70–1.67)	0.737
Male	1.20 (0.80–1.79)	0.377		
BMI	0.99 (0.95–1.03)	0.478		
Smoked	0.97 (0.63–1.48)	0.876		
FVC%	0.85 (0.80–0.90)	<0.001	0.85 (0.77–0.95)	0.003
DLCO%	0.73 (0.61–0.86)	<0.001	0.88 (0.70–1.11)	0.282
Cough VAS	1.07 (1.04–1.11)	<0.001	1.04 (0.98–1.11)	0.148
Dyspnea	1.93 (1.30–2.86)	0.001	1.24 (0.58–2.67)	0.575
Oxygen therapy	2.37 (1.53–3.66)	0.001	1.77 (0.86–3.63)	0.120

* In this model, we evaluated the impact of increasing age by 10 years, FVC% by 5%, DLCO% by 10%, and cough VAS by 5 units. Abbreviations: BMI, body mass index; DL_CO_, diffusing capacity of the lungs for carbon monoxide; FVC, forced vital capacity; UIP, usual interstitial pneumonia.

## Data Availability

The datasets used and/or analyzed during the current study are available from the corresponding author upon reasonable request.

## Supplementary Materials

The following supporting information can be downloaded at: https://www.mdpi.com/article/10.3390/jcm13237170/s1, Table S1: Baseline characteristics of patients with or without more than twice PFT examinations. Table S2: Comparison of baseline characteristics between patients with d-UIP and progressive p-UIP. Table S3: Comparison of baseline characteristics between patients with d-UIP & progressive p-UIP and stable p-UIP. Table S4: Risk factors associated with survival in patients with d-UIP & progressive p-UIP. Table S5: Subgroup analysis of changes in FVC and DLCO percent. Figure S1: The typical images of probable UIP and definite UIP on HRCT. Figure S2: Survival estimates of d-UIP and p-UIP in patients with and without smoking history. Figure S3: Survival estimates of d-UIP and p-UIP in male and female patients. Figure S4: The HRCT patterns for the progression of p-UIP. Figure S5: The differences between probable UIP and definite UIP.

## Abbreviations

6MWD = 6 min walking distance; BMI = body mass index; DL_CO_ = diffusing capacity of the lungs for carbon monoxide; d-UIP = definite usual interstitial pneumonia; FVC = forced vital capacity; HRCT = high-resolution chest computed tomography; IPF = idiopathic pulmonary fibrosis; p-UIP = probable usual interstitial pneumonia.
