# Supplementary material for: Differences Between Patients with Probable UIP and Definite UIP on HRCT in Idiopathic Pulmonary Fibrosis: A Real-World Cohort Study
Source: J Clin Med. 2024 Nov 26;13(23):7170. doi: 10.3390/jcm13237170 (PMC11642722; doi:10.3390/jcm13237170)

Supplemental Appendix to:

Differences between patients with probable UIP and definite UIP on HRCT in idiopathic pulmonary fibrosis: a real-world cohort study.

Chen et al.

Table S1. Baseline characteristics of patients with or without more than twice PFT examinations.

Table S2. Comparison of baseline characteristics between patients with d-UIP and progressive p-UIP

Table S3. Comparison of baseline characteristics between patients with d-UIP & progressive p-UIP and stable p-UIP.

Table S4. Risk factors associated with survival in patients with d-UIP & progressive p-UIP.

Table S5. Subgroup analysis of changes in FVC and DL_{CO} percent.

Figure S1. The typical images of probable UIP and definite UIP on HRCT.

Figure S2. Survival estimates of d-UIP and p-UIP in patients with and without smoking history

Figure S3. Survival estimates of d-UIP and p-UIP in male and female patients.

Figure S4. The HRCT patterns for the progression of p-UIP.

Figure S5. The differences between probable UIP and definite UIP.

Table S1. Baseline characteristics of patients with or without more than twice PFT examinations.

Variables	PFT >= twice	PFT < twice	P
Sample size	404	380	
Age, years	68±8	69±9	0.17
Male sex	295(73%)	283(74%)	0.64
BMI	28.5±4.8	28.2±5.3	0.49
Ever-smoker	290(73%)	292(78%)	0.07
Smoking pack-years	25(11-38)	25(11-40)	0.35
FVC, L	2.8±0.8	2.8±0.9	0.57
FVC, %-predicted	76±19	75±20	0.65
DLCO, %-predicted	55±18	51±17	0.03
6MWD	420±131	400±130	0.25
Resting oxygen saturation	97±2	96±3	<0.01
Exertional oxygen saturation	87.6±6.0	89.6±5.5	0.02
Oxygen therapy	102(28%)	73(25%)	0.30
Cough-Vas	25(10-48)	26(8-51)	0.88

Tabel S2. Comparison of baseline characteristics between patients with d-UIP and progressive p-UIP

Variables	d-UIP	Progressive p-UIP	P
Sample size	480	86	
Age, years	69±8	68±9	0.33
Male sex	363(76%)	58(67%)	0.11
BMI	28.4±5.0	28.7±5.5	0.59
Ever-smoker	380(80%)	55(66%)	0.01
Smoking pack-years	26[13-41]	20[7-37]	0.03
FVC, L	2.8±0.8	2.7±0.9	0.90
FVC, %-predicted	74±19	76±20	0.25
DLCO, %-predicted	50±16	58±18	<0.01
6MWD	410±131	402±154	0.75
Resting oxygen saturation	96.4±2.8	97.3±2.0	0.05
Exertional oxygen saturation	88.3±5.8	87.7±6.5	0.64
Oxygen therapy	102(27%)	33 (40%)	0.02
Cough-Vas	26[10-52]	36[16-50]	0.88
Dyspnea	261(54%)	48(56%)	0.81

Tabel S3. Comparison of baseline characteristics between patients with d-UIP & progressive p-UIP and stable p-UIP.

Variables	d-UIP & progressive p-UIP	stable p-UIP	P
Sample size	566	81	
Age, years	69±8	67±9	0.17
Male sex	421(74%)	57(70%)	0.44
BMI	28.4±5.1	27.8±4.2	0.30
Ever-smoker	435(78%)	51(65%)	0.01
Smoking pack-years	25[13-41]	25[13-36]	0.09
FVC, L	2.8±0.8	2.9±0.9	0.08
FVC, %-predicted	74±19	78±18	0.13
DLCO, %-predicted	52±17	63±18	<0.01
6MWD	409±135	465±100	0.05
Resting oxygen saturation	96.6±2.7	97.8±1.7	0.02
Exertional oxygen saturation	88.2±5.9	88.4±6.2	0.86
Oxygen therapy	135(29%)	16(20%)	0.08
Cough-Vas	28[11-51]	16[8-33]	<0.01

Table S4. Risk factors associated with survival in patients with d-UIP & progressive p-UIP.

Variables	Univariate*		Multivariate*	
	HR (95% CI)	*P*-value	HR (95% CI)	*P*-value
Age	0.97(0.79-1.17)	0.729	0.90(0.63-1.28)	0.553
Male	1.16(0.80-1.68)	0.427		
BMI	0.98(0.95-1.02)	0.357		
Smoked	1.06(0.72-1.56)	0.781		
FVC%	0.84(0.79-0.88)	<0.001	0.89(0.82-0.97)	0.005
DLCO%	0.69(0.59-0.80)	<0.001	0.79(0.65-0.95)	0.014
Cough Vas	1.09(1.05-1.12)	<0.001	1.04(0.98-1.09)	0.174
Dyspnea	1.97(1.38-2.82)	<0.001	1.25(0.65-2.41)	0.505
Oxygen therapy	2.74(1.84-4.07)	<0.001	1.65(0.89-3.07)	0.113

Table S5. Subgroup analysis of changes in FVC and DL_{CO} percent.

Variables	Changes in FVC%*		Changes in DL_{CO}%*	
	**Coefficient(95%CI)**	**P**	**Coefficient(95%CI)**	**P**
**Ever-smoker**	-0.18(-1.27, 0.92)	0.749	-0.13(-1.40, 1.14)	0.845
**Never-smoker**	0.65(-1.65, 2.95)	0.911	0.31(-2.28,2.90)	0.814
**Female**	-1.69(-3.32, -0.07)	0.041	-0.19(-2.37, 1.98)	0.862
**Male**	0.37(-0.83, 1.57)	0.545	-0.27(-1.62, 1.07)	0.691

*After adjusted with age, gender, baseline FVC and DL_{CO} and interval of PFT time, we evaluated the changes of FVC% and DL_{CO}% in p-UIP compared with d-UIP.

Figure S1. The typical images of probable UIP and definite UIP on HRCT.

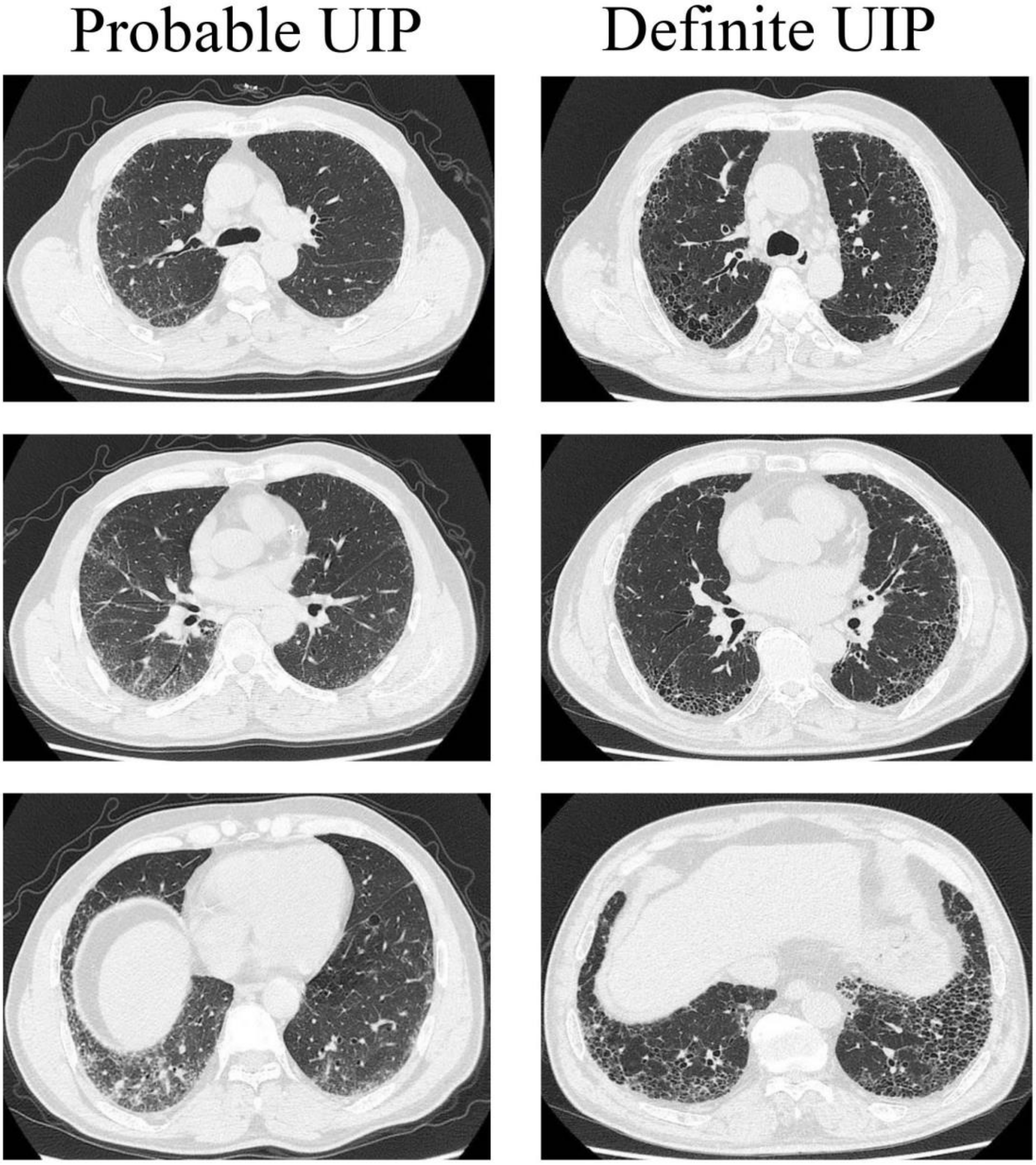

Figure S2. Survival estimates of d-UIP and p-UIP in patients with and without smoking history

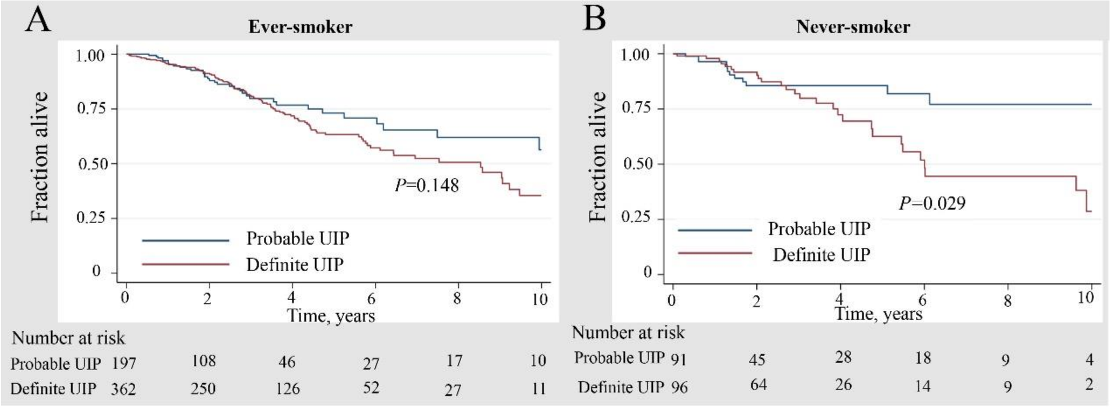

Figure S3. Survival estimates of d-UIP and p-UIP in male and female patients.

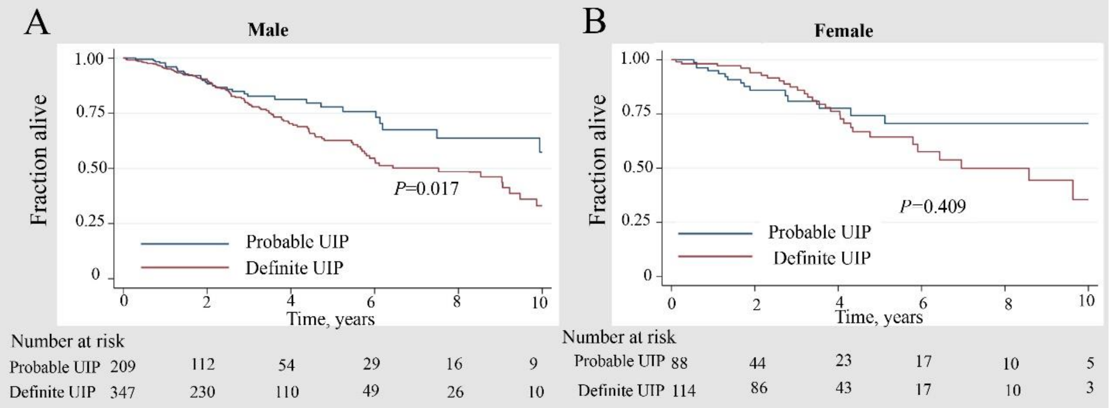

Figure S4. The HRCT patterns for the progression of p-UIP.

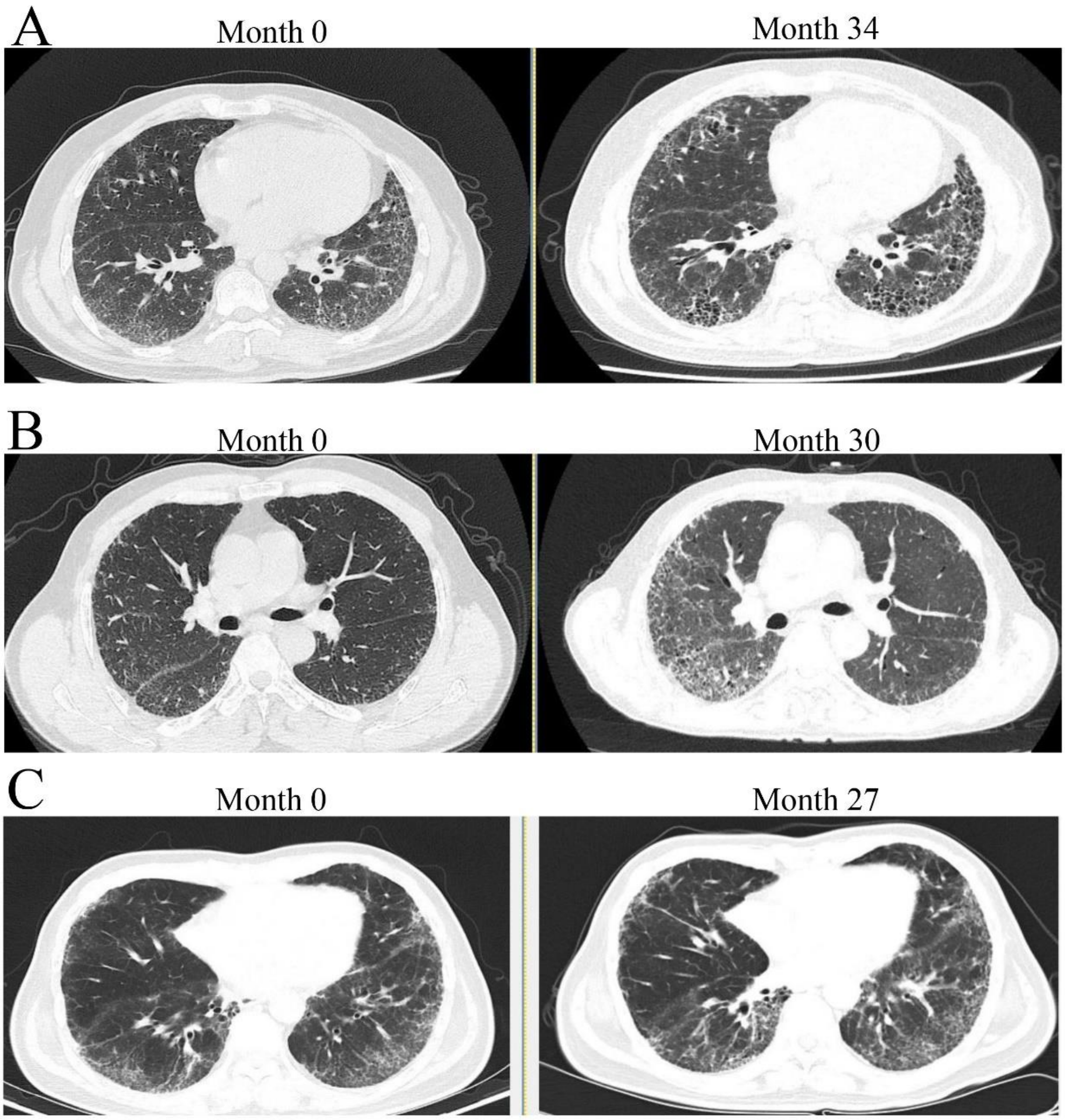

(A). The typical CT images for p-UIP progressed into d-UIP

(B). The typical CT images for progression of p-UIP with honeycombing formation in some areas.

(C). The typical CT images for progression of p-UIP with no honeycombing formation.

Figure S5. The differences between probable UIP and definite UIP.

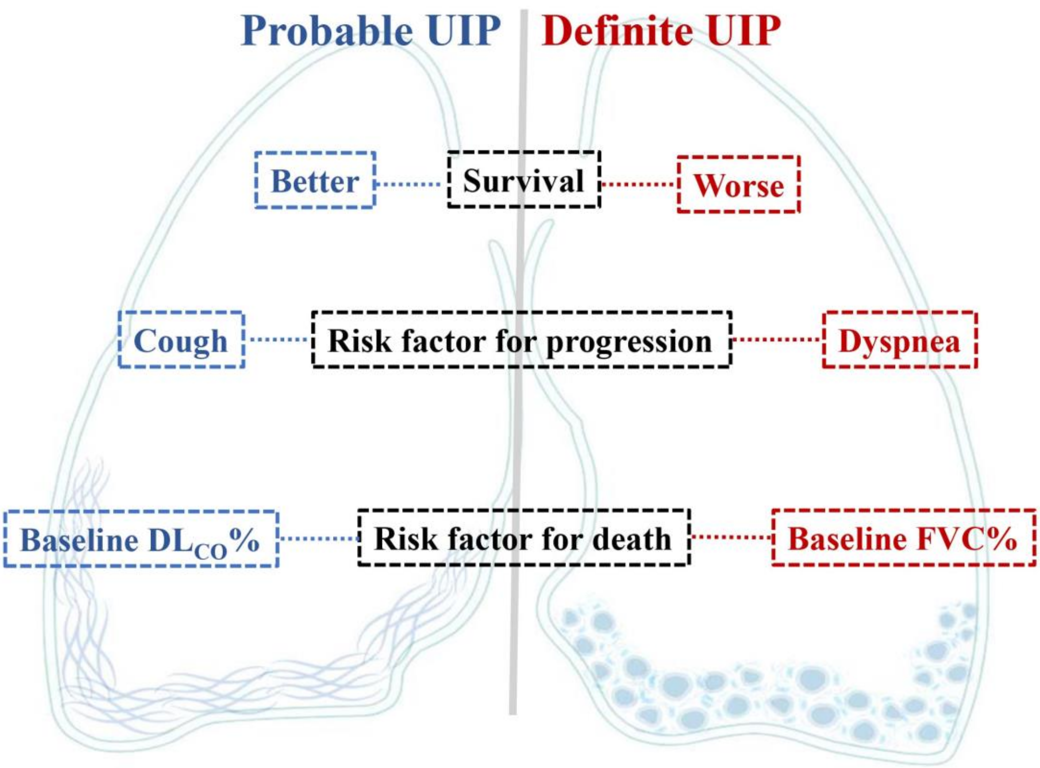

Supplement: Supplementary file 1 [file jcm-13-07170-s001.zip › jcm-3164073-supplementary.pdf]
